# Simultaneous resection of pituitary adenoma and clipping of aneurysm through endoscopic endonasal approach: a case report

**DOI:** 10.3389/fonc.2024.1341688

**Published:** 2024-05-24

**Authors:** Wenbo He, Chongxi Xu, Datong Zheng, Danyang Jie, Jianguo Xu, Songping Zheng

**Affiliations:** ^1^ Department of Neurosurgery, West China Hospital of Sichuan University, Chengdu, Sichuan, China; ^2^ West China School of Medicine, Sichuan University, Chengdu, China

**Keywords:** pituitary adenoma, intracranial aneurysm, endoscopic endonasal approach, clipping, surgical technique

## Abstract

Pituitary adenomas and intracranial aneurysms are prevalent neurosurgical conditions, but their simultaneous presence is uncommon, affecting only 0.5%-7.4% of those with pituitary adenomas. The strategy of treating aneurysms endovascularly before removing pituitary adenomas is widely adopted, yet reports on addressing both conditions at once through an endoscopic endonasal approach (EEA) are scarce. We present a case involving a pituitary adenoma coupled with an anterior communicating artery aneurysm. Utilizing the EEA, we excised the adenoma and clipped the aneurysm concurrently. The patient recovered well post-surgery, with follow-up assessments confirming the successful resolution of both the adenoma and aneurysm. We proved the feasibility of the EEA in the treatment of pituitary adenomas with anterior communicating artery aneurysms under specific anatomical relationships and close intraoperative monitoring.

## Introduction

Pituitary adenoma is a prevalent neurosurgical disease, with approximately 1 case per 1000 people in the general population (1). Removal of pituitary adenomas by the endoscopic endonasal approach (EEA) has become a mature method. However, pituitary adenoma with an aneurysm is a rare case, with an incidence of approximately 0.5% and 7.4% (2–5). Generally, if there are aneurysms in close contact with pituitary adenomas, endoscopic resection cannot be performed because this may lead to ruptured aneurysms with severe consequences (6–8). For this special case, there was preoperative endovascular securing of the aneurysm, endoscopic pituitary adenoma resection combined with aneurysm clipping by craniotomy and other complex methods. We report a special case of pituitary adenoma with an unruptured anterior communicating artery aneurysm. We performed simultaneous resection of the pituitary adenoma and clipping of the aneurysm under nasal endoscopy, which confirmed the feasibility and advantages of this method under specific anatomical conditions and close intraoperative monitoring. The related literature was reviewed, and it was pointed out that it was quite rare in the world to deal with two kinds of lesions at the same time through EEA.

## Case illustration

### Initial presentation

**Figure 1:**
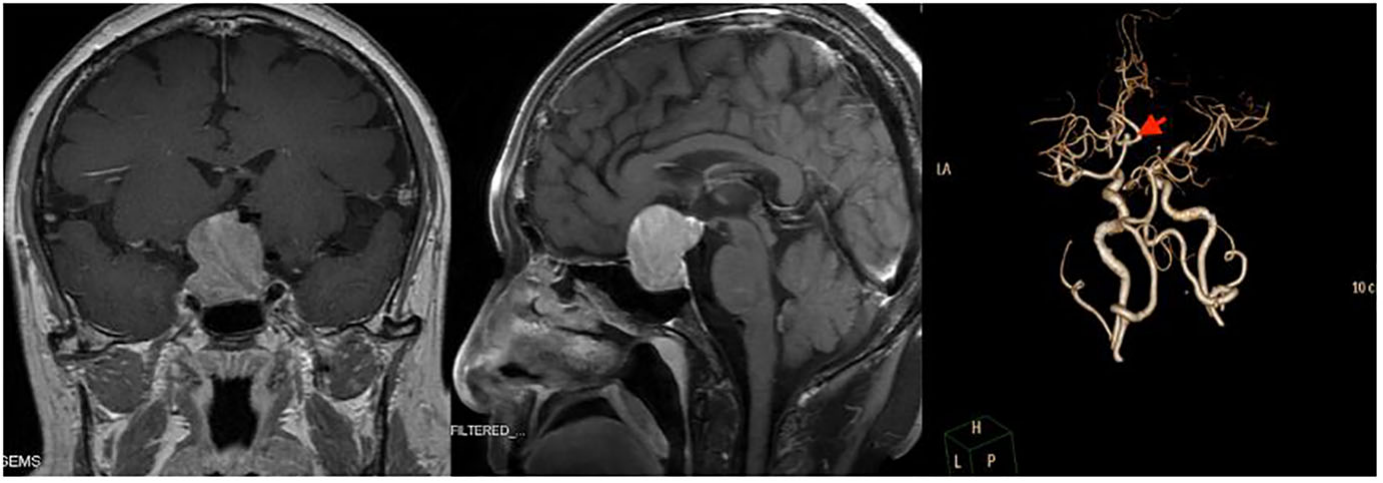
Preoperative imaging examination of the reported patient. Left and Middle: Sagittal and coronal T1 MR imaging after injection of gadolinium. Both pictures showed massive masses in the sellar fossa and suprasellar cistern. Right: Computed Tomography Angiography (CTA) showed an aneurysm at the anterior edge of the anterior communicating artery, with a diameter of about 0.5cm.

A 56-year-old Chinese man sought treatment at our institution, West China Hospital, for a residual pituitary adenoma. One year previously, the patient was admitted to a local hospital for headaches and diminished visual acuity in his right eye. Pituitary magnetic resonance imaging (MRI) showed solid space-occupying lesions in the sellar and suprasellar regions, measuring about 2.6*2.6*1.9 cm, which compressed the optic chiasm. Additionally, a magnetic resonance angiography (MRA) of the head uncovered an aneurysm in the anterior cerebral artery, sized roughly 0.4 x 0.5 cm. The local hospital carried out a transsphenoidal surgery to remove the mass in the sellar region, but only about one-third of the lesion was excised due to the aneurysm. A postoperative biopsy confirmed the mass as a pituitary adenoma. A year later, a follow-up MRI at the same local hospital revealed a residual pituitary adenoma with hemorrhage, approximately 2.2 cm in diameter. To address the residual tumor, the patient was admitted to our institution for further treatment. Upon admission, the patient complained of occasional headache, dizziness and slight vision impairment. A visual acuity test revealed reduced vision in both eyes (OD 0.5, OS 0.6). Hormonal tests indicated a significant decrease in thyroid and sex hormones, including testosterone and estradiol, while follicle-stimulating hormone (FSH) showed a mild increase (15.01 U/L). Pituitary enhanced MRI showed enlarged mass in the sellar fossa and suprasellar cistern, with a size of approximately 2.7*3.1*2.9 cm. The enhanced scan showed heterogeneous enhancement, and the bilateral cavernous sinus was involved. Computed tomography angiography (CTA) showed that the right anterior A1 segment of the brain was absent, and an aneurysm (neck: 0.3cm, dome: 0.5cm) was seen at the anterior edge of the anterior communicating artery. Detailed preoperative imaging is presented in **Figure 1**.

### Preoperative risk analysis and surgical plan

The highest risk associated with this surgery is the rupture of the aneurysm during the procedure. In this regard, we performed detailed preoperative imaging studies, which showed that the anterior communicating aneurysm was small and projected superiorly. Endoscopic clipping of aneurysms in such locations and sizes is practical. Considering the patient’s financial situation, willingness to undergo craniotomy, and adherence to postoperative care, we advise opting for the Endoscopic Endonasal Approach (EEA) to concurrently address the pituitary adenoma and clip the aneurysm. Should the aneurysm prove challenging to expose or clip during surgery, a swift transition to craniotomy is recommended.

### Surgical procedure

**Figure 2:**
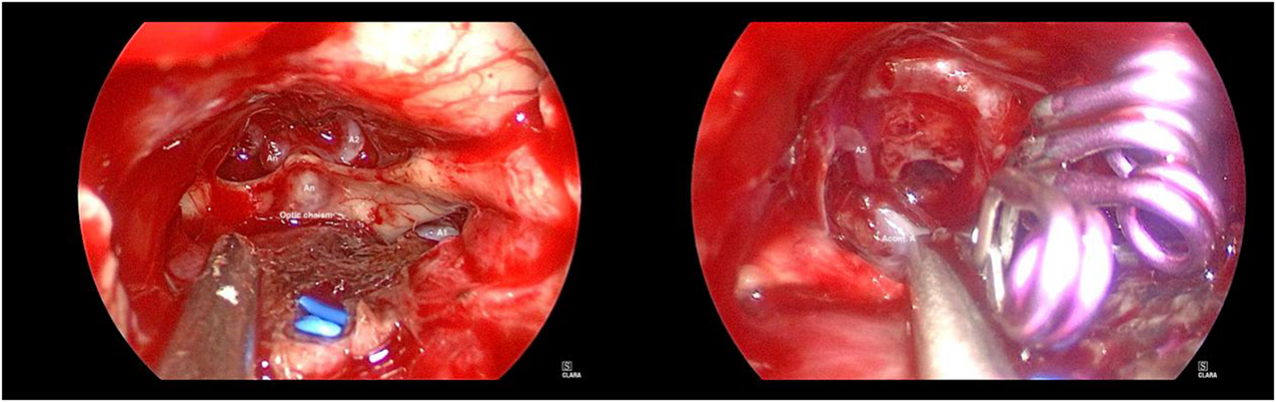
Intraoperative nasal endoscopic images of the reported patient. Left: After resection of sellar region masses and opening of dura mater, it can be seen that the aneurysm was located in the anterior communicating artery. Right: The aneurysm neck was clamped with 3 aneurysm clips and the bilateral A2 filled well.

After general anaesthesia, the patient was placed in the supine position with the head in the middle position and leaned back 15 degrees. After preparation, the extended transnasal approach was adopted. The bilateral superior turbinate and middle and lower parts of the middle turbinate were resected under nasal endoscopy. Then, part of the bilateral posterior ethmoid sinus and the posterior bone and mucosa of the nasal septum were removed. Next, we removed the forearm of the sphenoid sinus and the mucous membrane of the sphenoid sinus and then ground the bone of the sellar floor, tuberculum sellae and anterior skull base after disinfection. After incision of the dura mater of the sellar floor, the lesion was found to be located in the sellar region. The lesion was dark red and soft, with abundant blood supply, pushing upwards and compressing the normal pituitary, bilateral optic nerve and optic chiasma. After the lesion was removed with a curette and tumor forceps, the septum sellae was sunken. Then, we opened the dura mater of the tubercle sellae and anterior skull base. After separating the adhesion between the sellar septum and the optic nerve and the arachnoid space of the optic chiasma, the dominant blood supply of the A1 segment of the left internal carotid artery could be seen. An aneurysm was found in the anterior communicating artery, approximately 0.5 cm*0.5 cm*0.5 cm dome size, and the neck was approximately 0.3 cm. The left A1 was temporarily clipped, and the aneurysm neck was clamped with 3 aneurysm clips. Finally, the bilateral A2 filled well, indicating that the anterior communicating artery was not mistakenly clamped. The intraoperative nasal endoscopic images are shown in **Figure 2**.

### Repair strategy of the sellar floor

After achieving complete hemostasis, the fascia lata from the right lower limb was harvested, and the skull base repair utilized artificial dura mater, fascia lata, a right nasal septum mucous membrane flap, and fibrin glue. Subsequently, two strips of iodoform gauze and absorbent cotton were positioned in the nasal cavity to bolster the repair materials. The reconstruction of the sellar floor was ultimately successful.

### Intraoperative neurophysiological monitoring

We used intraoperative neurophysiological monitoring (IONM), including limb somatosensory evoked potential (SEP), transcranial motor evoked potential (MEP), electromyography (EMG), electroencephalogram (EEG), and train-of-four ratio (TOF). The findings indicate that after completely occluding the A1 segment of the left anterior cerebral artery for 41 minutes, there were no significant alterations in SEP and MEP. Similarly, no substantial changes were observed in limb SEP and MEP post-surgery, and both EEG and EMG remained normal throughout the procedure.

### Postoperative management

**Figure 3:**
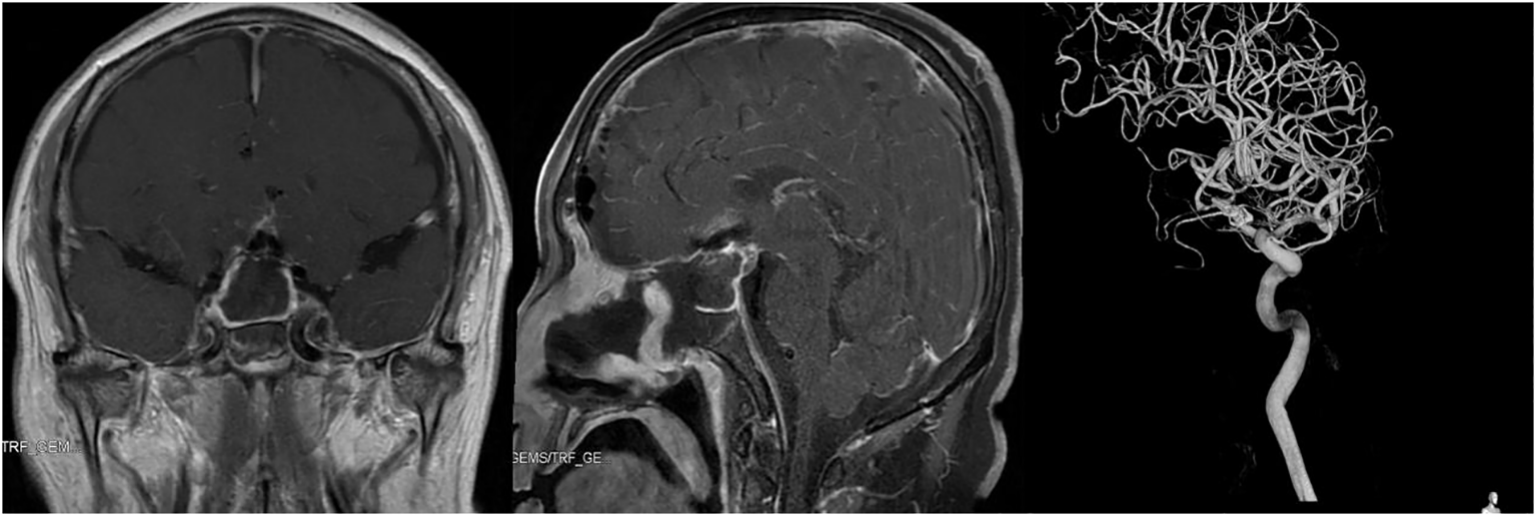
Imaging examination of the reported patient after operation. Left and Middle: Pituitary enhanced MRI showed complete removal of sellar region lesion 3 days after operation. Right: Catheter-based cerebral angiography indicated no obvious abnormality after clipping of anterior communicating artery aneurysm.

After the operation, the patient received treatment such as infection and seizure prophylaxis, hormone supplementation, and maintenance of fluid and electrolyte balance. The results of CTA 1 day after the operation showed that the aneurysm was well clipped, and pituitary enhanced MRI showed complete removal of the sellar region lesion 3 days after the operation (**Figure 3**). In addition, one week after the operation, catheter-based cerebral angiography indicated no obvious abnormality after clipping of the anterior communicating artery aneurysm. The postoperative pathological biopsy confirmed a pituitary adenoma with FSH secretion, which, when taken together with clinical assessments, was consistent with a nonfunctional pituitary adenoma diagnosis. The patient experienced no serious complications during the hospital stay, including cerebrospinal fluid leakage, cerebral hemorrhage, or cerebral infarction. The patient developed an intracranial infection, and the cerebrospinal fluid test returned to normal after treatment with vancomycin combined with meropenem. In addition, transient central diabetes insipidus occurred and improved after treatment with desmopressin acetate. At discharge, the patient recovered well, without headache, nasal exudation, loss of vision, disturbance of eye movement, ptosis or other neurological impairment.

### Follow-up

**Figure 4:**
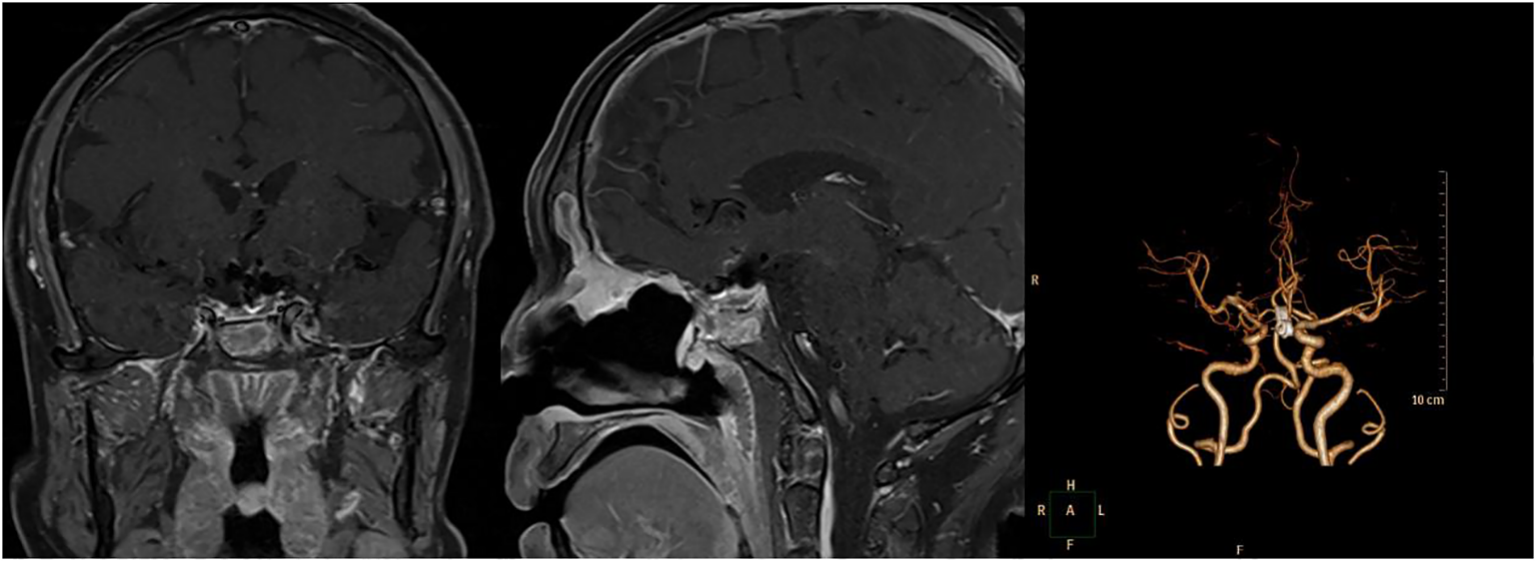
The detailed images reexamined 6 months after operation. Left and Middle: Pituitary enhanced MRI showed no recurrence of the pituitary adenoma 6 months after operation. Right: Computed Tomography Angiography (CTA) 6 months after operation showed the aneurysm was well clipped.

The patient underwent a follow-up examination at our hospital six months post-operation. Throughout this period, the patient exhibited no symptoms, including dizziness, headaches, reduced vision, nasal discharge, or changes in urine output. Furthermore, the neurological examination revealed no noteworthy findings. and no positive signs in the nervous system examination. Pituitary MRI scans indicated no evident signs of tumor recurrence, and the CTA confirmed the aneurysm was successfully clipped. **Figure 4** presents the imaging taken six months following the surgery.

## Discussion

### Incidence

Pituitary adenomas and intracranial aneurysms are common neurosurgical diseases, but the incidence of pituitary adenomas complicated with intracranial aneurysms is low. With the development of angiography and imaging technology, an increasing number of incidental aneurysms have been found. A recent retrospective study of a large sample of 800 pituitary adenomas revealed that the incidence of pituitary adenomas with aneurysms was 2.3% (2). In addition, several previous series have reported such a situation, with an incidence of 0.5%-7.4% (2–5), which is higher than the incidence of aneurysms with other brain neoplasms (3). Internal carotid artery and anterior communicating artery aneurysms are more likely to occur in patients with pituitary adenomas because they are the main blood supply of the pituitary region (9). In a retrospective study of 467 cases of pituitary adenomas, 60% of aneurysms occurred near the pituitary region, and 40% of aneurysms occurred distally (10). This may be due to direct compression of blood vessels by pituitary adenomas, changes in cerebral circulation caused by tumor accumulation in the skull base, or growth hormone secretion that leads to arteriosclerosis and degenerative changes in the walls of the Willis ring arteries (11).

### Surgical strategy

Sometimes the indication for the treatment of unruptured aneurysms is to remove the adjacent lesion. Adjacent unruptured aneurysms may rupture during surgery when dealing with these lesions, which is very dangerous in sellar region surgery (6–8). Lesions in the sellar region are usually resected by the transnasal transsphenoidal approach (TSS). A retrospective study showed that the mortality rate after carotid artery injury during transnasal surgery was 14%, and 24% of people developed severe neurological impairment (12). If the aneurysm is in close contact with the lesions in the sellar region, the surgical strategy will become complicated.

The complication rate of endovascular therapy is lower than that of craniotomy, so endovascular therapy before resection of pituitary adenomas is often the first choice for many institutions to treat these complex diseases (11–14). However, the problem with endovascular therapy is the need for long-term antiplatelet drugs, which may delay the treatment of pituitary apoplexy, especially if the patient already has vision loss or pituitary stroke. Endovascular coiling usually does not require the use of antiplatelet drugs after the operation, but it is highly dependent on the shape of the aneurysm and may lead to incomplete disappearance or recurrence of the aneurysm (15).

To avoid long-term use of antiplatelet drugs, other treatments must be considered. Several surgical strategies have been reported in the past. The simultaneous use of two treatments via a transcranial approach is a classic treatment strategy that has been adopted by some institutions, but craniotomy has disadvantages compared with the EEA (16, 17). EEA is the most direct way to deal with median ventral lesions of the skull base, which is often easier than craniotomy to remove lesions in the sellar region while avoiding brain contraction and minimizing the operation of key neurovascular structures. The multicentric series conducted by Chibbaro et al. have confirmed the effectiveness and safety of EEA in pituitary adenomas, and confirmed the flexibility of the wide surgical corridor provided by EEA in treating ventral lesions of the craniocervical junction (18, 19). As an improvement, some institutions use the EEA to deal with sellar lesions and clip aneurysms by craniotomy (5). However, this approach produces an additional surgical incision, accompanied by a higher rate of complications and surgical costs. Another surgical strategy is to deal with pituitary adenomas and aneurysms by the EEA at the same time. Endoscopic resection of pituitary adenomas is a very mature technique, and endoscopic clipping of aneurysms has recently been proven to be feasible by many institutions (20, 21). In specific cases, intracranial aneurysms can be removed by TSS as long as the basic principles of cerebrovascular surgery are respected (7). Recent anatomical studies have demonstrated that resection of anterior communicating artery aneurysms and A1 and A2 aneurysms may be feasible, but this method should only be adopted in anterior communicating artery aneurysm aneurysms (22). Anterior communicating artery aneurysms can successfully expose A1, but not all specimens can expose A2 (23, 24). At present, there are institutions that deal with both Rathke cleft cysts and aneurysms through the EEA. Similarly, we have treated both pituitary adenomas and aneurysms through EEA simultaneously.

### Experience

First, endovascular therapy before resection of pituitary adenomas is the first choice in most cases. However, in a team with experience in EEA and cerebrovascular surgery, as well as adequate instruments and monitoring equipment, endoscopic personalized treatment of aneurysms combined with pituitary adenomas at specific locations is no longer a contraindication to surgery. Second, the extended transsphenoidal approach can be used when pituitary adenomas are complicated with specific aneurysms. Due to the limitations of nasal structure and angle, endoscopic treatment of aneurysms is more suitable for aneurysms with medial or medial superior protuberance of the superior clinoid segment of the internal carotid artery or medial and ventral aneurysms of the anterior communicating artery. Third, endoscopic treatment of unruptured aneurysms is more suitable because removal of pituitary adenomas may cause unstable ruptured aneurysms to rupture again, which is difficult to address by nasal endoscopy. Fourth, it is necessary to remove part of the pituitary adenomas before so that the anterior communicating artery aneurysm pushed upwards can be moved downwards, making clipping possible. Fifth, after partial resection of pituitary adenomas, searching for the A1 segment of the internal carotid artery for early proximal control is an important guarantee for surgical safety. Sixth, endoscopic clipping of aneurysms can reduce the additional trauma caused by multiple operations, save costs, and promote patient’s rapid postoperative recovery. Finally, when pituitary adenoma is complicated with ruptured aneurysm, endovascular treatment or craniotomy is recommended to treat the aneurysm before pituitary adenoma.

## Conclusion

Under the specific anatomical relationship, simultaneous treatment of pituitary adenomas with unruptured aneurysms by EEA is a feasible method and can reduce postoperative complications and treatment costs.

## Data availability statement

The original contributions presented in the study are included in the article/supplementary material. Further inquiries can be directed to the corresponding author.

## Ethics statement

The studies involving humans were approved by West-China Hospital Research Ethics Committee. The studies were conducted in accordance with the local legislation and institutional requirements. Written informed consent for participation was not required from the participants or the participants’ legal guardians/next of kin in accordance with the national legislation and institutional requirements. Written informed consent was obtained from the individual(s) for the publication of any potentially identifiable images or data included in this article.

## Author contributions

WH: Conceptualization, Data curation, Methodology, Visualization, Writing – original draft, Writing – review & editing. CX: Conceptualization, Data curation, Methodology, Writing – original draft. DZ: Conceptualization, Data curation, Methodology, Writing – original draft. DJ: Data curation, Methodology, Writing – original draft. JX: Conceptualization, Supervision, Writing – review & editing. SZ: Conceptualization, Funding acquisition, Project administration, Supervision, Validation, Writing – review & editing.
